# *Echinoderes pterus* sp. n. showing a geographically and bathymetrically wide distribution pattern on seamounts and on the deep-sea floor in the Arctic Ocean, Atlantic Ocean, and the Mediterranean Sea (Kinorhyncha, Cyclorhagida)

**DOI:** 10.3897/zookeys.771.25534

**Published:** 2018-07-05

**Authors:** Hiroshi Yamasaki, Katarzyna Grzelak, Martin V. Sørensen, Birger Neuhaus, Kai Horst George

**Affiliations:** 1 Museum für Naturkunde, Leibniz Institute for Evolution and Biodiversity, Invalidenstr. 43, D-10115 Berlin, Germany; 2 Senckenberg am Meer, Abt. Deutsches Zentrum für Marine Biodiversitätsforschung DZMB, Südstrand 44, D-26382 Wilhelmshaven, Germany; 3 Laboratory of Polar Biology and Oceanobiology, Faculty of Biology and Environmental Protection, University of Łódź, Łódź, Poland; 4 Polish Academy of Sciences, Institute of Oceanology, Sopot, Poland; 5 Natural History Museum of Denmark, University of Copenhagen, Copenhagen, Denmark

**Keywords:** meiofauna, meiofauna paradox, morphological variation, taxonomy

## Abstract

Kinorhynchs rarely show a wide distribution pattern, due to their putatively low dispersal capabilities and/or limited sampling efforts. In this study, a new kinorhynch species is described, *Echinoderes pterus* **sp. n.**, which shows a geographically and bathymetrically wide distribution, occurring on the Karasik Seamount and off the Svalbard Islands (Arctic Ocean), on the Sedlo Seamount (northeast Atlantic Ocean), and on the deep-sea floor off Crete and on the Anaximenes Seamount (Mediterranean Sea), at a depth range of 675–4,403 m. The new species is characterized by a combination of middorsal acicular spines on segments 4–8, laterodorsal tubes on segment 10, lateroventral tubes on segment 5, lateroventral acicular spines on segments 6–9, tufts of long hairs rising from slits in a laterodorsal position on segment 9, truncated tergal extensions on segment 11, and the absence of any type-2 gland cell outlet. The specimens belonging to the populations from the Arctic Ocean, the Sedlo Seamount, and the Mediterranean Sea show morphological variation in the thickness and length of the spines as well as in the presence/absence of ventromedial sensory spots on segment 7. The different populations are regarded as belonging to a single species because of their overlapping variable characters.

## Introduction

The meiofauna, defined as the assemblage of microscopic benthic organisms passing through a 1 mm-sieve mesh and collected on a 40–63 µm-sieve mesh, is composed of various taxonomic groups, and occurs in diverse habitats including extreme environments such as polar regions, the deep sea, and seamounts (George 2013; De Broyer et al. 2014; Zeppilli et al. 2018). While meiobenthic organisms are generally thought to have a low dispersal ability because of their low mobility as well as their lack of a planktonic larval stage, some meiofaunal species can show a wide distribution pattern. This phenomenon is referred to the “meiofauna paradox” or “everything is everywhere hypothesis” (Giere 2009; Fontaneto 2011). Such wide distribution patterns have been explained or hypothesized by the stepping stone hypothesis (George 2013; Packmor and Riedl 2016), artificial dispersal (artificial invasion) (Herranz and Leander 2016; Pardos et al. 2016; Cvitković et al. 2017), or long range dispersal using currents and/or drifting (Walters and Bell 1994; Neuhaus and Sørensen 2013; Neuhaus et al. 2014; Yamasaki et al. 2014). Some are even regarded as a pseudo-wide distribution via the detection of cryptic species (Jörger et al. 2012; Leasi et al. 2016).

Kinorhyncha is an ecdysozoan phylum which is exclusively composed of marine meiofaunal species. To date, more than 260 kinorhynchs species are known from around the world (Grzelak and Sørensen 2018a, b; Yamasaki et al. 2018). Many ecological studies on meiofauna from various regions and environments often report the presence of Kinorhyncha, but unfortunately provide only phylum-level identification (e.g., Grzelak and Kotwicki 2012; Nomaki et al. 2016; Riera et al. 2018). Most kinorhynch species have been recorded from a single or few localities within a limited region only, probably due to their low dispersal ability like other meiofaunal organisms, but most likely also because of limited sampling activities. So far, only few kinorhynch species have been recorded as geographically wide distributed species either from both shallow waters and the deep sea, e.g., Campyloderes cf. vanhoeffeni Zelinka, 1913, or from several shallow-water stations interrupted by the deep sea, e.g., *Centroderes barbanigra* Neuhaus et al., 2014, *Echinoderes ohtsukai* Yamasaki & Kajiraha, 2012, and *Echinoderes tchefouensis* Lou, 1934 (Sørensen et al. 2012b, 2016; Neuhaus et al. 2014; Herranz and Leander 2016).

In the present study, we describe a new kinorhynch species with a geographically and bathymetrically wide distribution, ranging from the Arctic Ocean to the Mediterranean Sea and from upper bathyal to lower abyssal depths. The interpopulational morphological variation of the new species is also discussed.

## Materials and methods

**Figure 1.**
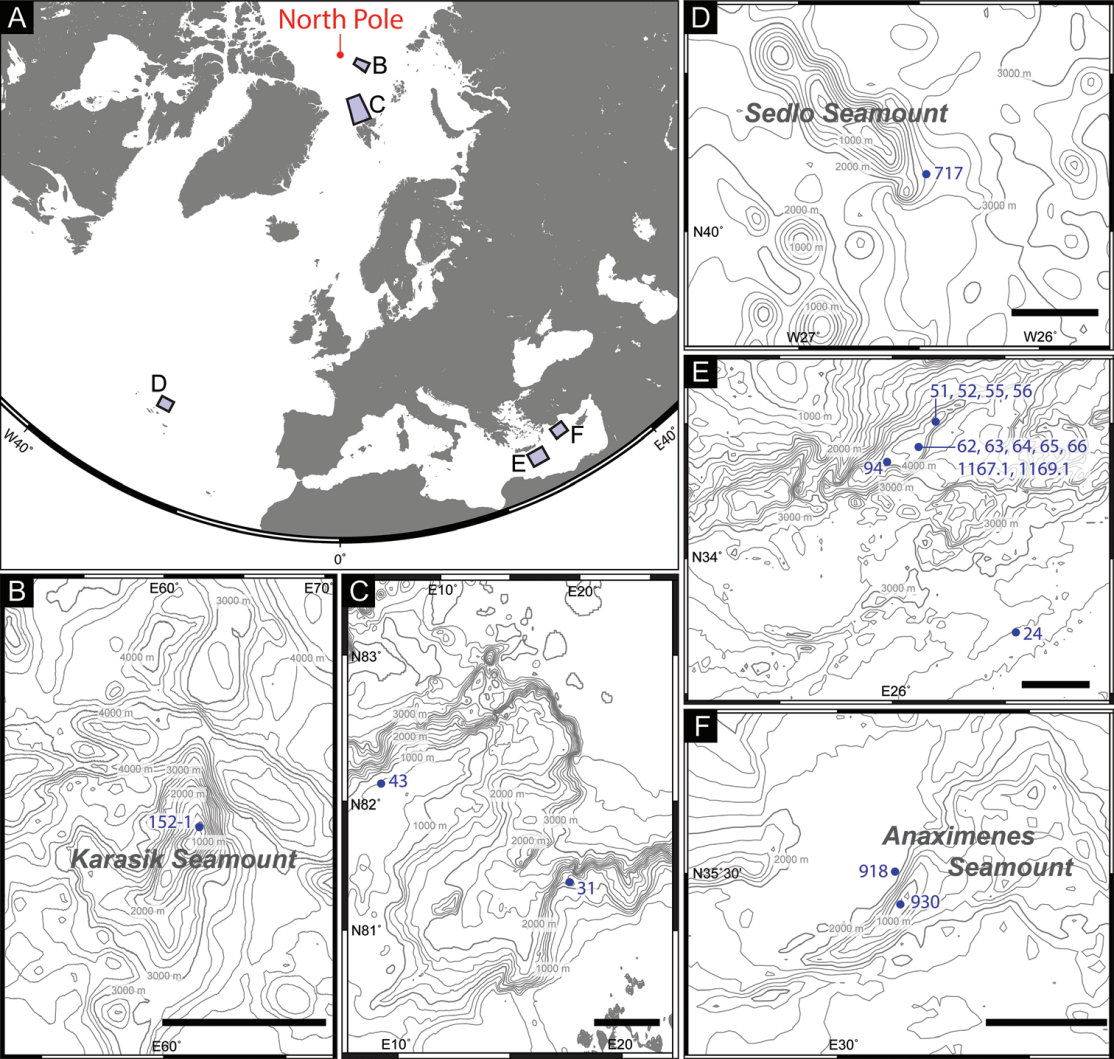
Map of the sampling localities. **A** map of the Northern hemisphere, including the Arctic Ocean, Atlantic Ocean, and Mediterranean Sea **B** enlarged map of the Karasik Seamount **C** enlarged map of the north of Svalbard **D** enlarged map of the Sedlo Seamount **E** enlarged map of the deep-sea canyon off Crete in the Mediterranean Sea **F** enlarged map of the Anaximenes Seamount. Scale bars: 20 km (**B**), 50 km (**C**), 30 km (**D–F**).

Kinorhynchs were obtained from meiofauna samples collected from the central mount of the Karasik Seamount, Langseth Ridge in the Arctic Ocean (by the R/V *Polarstern* during the expedition PS101, Boetius and Purser (2017)), north of Svalbard in the Arctic Ocean (by the R/V *Polarstern* during the expedition PS92, Peeken (2016)), on the Sedlo Seamount in the Atlantic Ocean (by the R/V *METEOR* during the expedition M60/1, Christiansen and Wolff (2009)), in a deep-sea trench off Crete and on the adjacent deep-sea floor in the Mediterranean Sea (by the R/V *METEOR* during the expedition M71/2, Christiansen et al. (2015), and R/V *Maria S. Merian* during the expedition MSM14/1, Christiansen et al. (2012)), and on the Anaximenes Seamount in the Mediterranean Sea (by the R/V *METEOR* during the expedition M71/1, Denda and Christiansen (2011)) (Fig. 1, Table 1). All sediment samples were fixed in 4–8% formaldehyde. Subsequently, the samples were washed with tap water on a 32-µm or a 40-µm mesh sieve in the laboratory, and the meiofauna was extracted from the sediment by centrifuging with a colloidal silica polymer (H.C. Stark, Levasil 200/40%, density 1.17 g/cm^3^) and Kaolin, or with a colloidal silica polymer (Ludox TS50, density 1.4 g/cm^3^). After extraction, the meiofauna was rinsed with tap water, sorted under a stereomicroscope, and subsequently preserved in 75% ethanol or 4% formaldehyde solution. Specimens collected during the expeditions PS92 and MSM14/1 were stained with Rose Bengal before sorting.

**Table 1.** Data on sampling stations.

Sampling region	Station	Cruise	Date	Depth [m]	Latitude	Longitude	Gear
Langseth Ridge, central mount on Karasik Seamount	152-1	PS101	28.09.2016	903	86°49'23"N, 61°40'10"E		multicorer
North of Svalbard	31	PS92	04.06.2015	1,656	81°28'11"N, 18°10'27"E		box corer
North of Svalbard	43	PS92	15.06.2015	790	82°12'19"N, 7°38'4"E		box corer
Sedlo Seamount	717	M60/1	24.11.2003	2,721	40°11'00"N, 26°33'6"W		box corer
Mediterranean deep sea	24	M71/2	04.01.2007	2,789	33°43'41"N, 26°32'55"E		multicorer
Mediterranean deep sea	51	M71/2	07.01.2007	4,323	34°30'19"N, 26°11'30"E		multicorer
Mediterranean deep sea	52	M71/2	07.01.2007	4,326	34°30'18"N, 26°11'31"E		multicorer
Mediterranean deep sea	55	M71/2	07.01.2007	4,332	34°30'19"N, 26°11'30"E		multicorer
Mediterranean deep sea	56	M71/2	08.01.2007	4,327	34°30'19"N, 26°11'31"E		multicorer
Mediterranean deep sea	62	M71/2	08.01.2007	4,396	34°25'5"N, 26°7'6"E		multicorer
Mediterranean deep sea	63	M71/2	08.01.2007	4,395	34°24'56"N, 26°6'59"E		multicorer
Mediterranean deep sea	64	M71/2	08.01.2007	4,399	34°24'59"N, 26°6'57"E		multicorer
Mediterranean deep sea	65	M71/2	09.01.2007	4,403	34°25'00"N, 26°6'59"E		multicorer
Mediterranean deep sea	66	M71/2	09.01.2007	4,401	34°25'00"N, 26°6'59"E		multicorer
Mediterranean deep sea	94	M71/2	12.01.2007	4,147	34°21'29"N, 25°58'30"E		multicorer
Mediterranean deep sea	1167.1	MSM14/1	11.01.2010	4,353	34°24'36"N, 26°7'31"E		multicorer
Mediterranean deep sea	1169.1	MSM14/1	12.01.2010	4,344	34°24'34"N, 26°7'30"E		multicorer
Anaximenes Seamount	918	M71/1	17.12.2006	2,043	35°30'14"N, 30°8'58"E		multicorer
Anaximenes Seamount	930	M71/1	19.12.2006	675	35°26'4"N, 30°9'53"E		multicorer

Specimens for light microscopy (LM) were dehydrated in glycerol and mounted as glycerol-paraffin slides on Cobb aluminum frames or mounted in Fluoromount G™ between two cover slips attached to a plastic H-S slide. LM specimens were observed with a Zeiss Axioskop 50 microscope, or with an Olympus BX51 microscope, and a Nikon E600 microscope. All microscopes were equipped with Nomarski differential interference contrast. A camera lucida equipped with a Zeiss Axioskop 50 microscope was used to make drafts for line art illustrations. Final line art illustrations were drawn with Adobe Illustrator CS6 based on the drafts. Measurements were made through a camera lucida or with Cell^D software. Specimens were photographed with a Zeiss AxioCam MRc5 or an Olympus DP27 camera.

Five specimens from the Karasik Seamount and 23 specimens from the Mediterranean deep sea were used for scanning electron microscopy (SEM) observation. The specimens were transferred from ethanol to distilled water through a graded series of ethanol, postfixed with OsO_4_ in 0.05 M phosphate buffer (pH = 7.3) with 0.3 M sodium chloride and 0.05% sodium azide for 2.5 hours, dehydrated through a graded series of ethanol, critical-point dried with a BalTec CPD 030, mounted on aluminum stubs, sputter-coated with gold-palladium with a Polaron SC 7640, and observed with a Zeiss EVO LS 10 scanning electron microscope.

The terminology follows Neuhaus and Higgins (2002), Sørensen and Pardos (2008) and Neuhaus (2013). All specimens, except those from Svalbard, have been deposited in the Museum für Naturkunde Berlin (= ZMB, former Zoological Museum Berlin), Germany, and catalogued in the collection “Vermes” in the “Generalkatalog Freilebende Würmer”. Specimens from Svalbard were deposited in the Natural History Museum of Denmark (NHMD). The maps of the sampling localities are drawn by the Generic Mapping Tools (GMT, https://www.soest.hawaii.edu/gmt/) using bathymetric data from the database of the National Center for Environmental Information.

## Results

### Taxonomy

#### Class Cyclorhagida Sørensen et al., 2015

##### Order Echinorhagata Sørensen et al., 2015

###### Family Echinoderidae Zelinka, 1894

####### Genus *Echinoderes* Claparède, 1863

######## Echinoderes pterus sp. n.

Taxon classificationAnimaliaEchinorhagataEchinoderidae

http://zoobank.org/7F59E70B-3B53-4168-929B-F0EDCB6CD231

Figs 2 , 3 , 4 , 5 , 6 , 7 , 8 , 9 ; Tables 2 and 3

######### Diagnosis.

*Echinoderes* with middorsal acicular spines on segments 4–8; laterodorsal tubes on segment 10; lateroventral tubes on segment 5; lateroventral acicular spines on segments 6–9; tufts of long hairs arising from slits in a laterodorsal position on segment 9; truncated tergal extensions on segment 11; without type-2 gland cell outlet.

######### Etymology.

The species name is derived from the Latinized Greek *pterón* (wing or feather), referring to the tufts of hairs on segment 9 which look like wings.

######### Material examined.

Holotype: Adult male (ZMB 11608), collected at station 55 in the Mediterranean deep sea off Crete (Fig. 1A, E; Table 1), mounted as a glycerol-paraffin slide on a Cobb aluminum frame.

Paratypes: Adults, collected in the Mediterranean Sea off Crete; four males and one female, collected at station 24 (ZMB 11609–11613); one female, collected at station 51 (ZMB 11614); one female, collected at station 52 (ZMB 11615); one male, collected at station 56 (ZMB 11616); one male and three females, collected at station 62 (ZMB 11617–11620); one female, collected at station 64 (ZMB 11621); one male, collected at station 65 (ZMB 11622); one male, collected at station 66 (ZMB 11623); one male, collected at station 94 (ZMB 11624); four males and seven females, collected at station 1167.1 (ZMB 11628–11638); one male and one female, collected at station 1169.1 (ZMB 11639–11640); one female, collected at station 918 (ZMB 11625); one male and one female, collected at station 930 (ZMB 11626–11627); (Fig. 1A, E, F; Table 1). All paratypes mounted as glycerol-paraffin slides on Cobb aluminum frames.

Additional material for LM: all adults; seven males and 12 females, collected at station 152 on Karasik Seamount, mounted as glycerol-paraffin slides on Cobb aluminum frames (ZMB 11642–11660); one male and one female, collected at station 31 north of Svalbard, mounted in Fluoromount G (NHMD-202798 and NHMD-202799); one male and one female, collected at station 43 north of Svalbard, mounted in Fluoromount G (NHMD-202800 and NHMD-202801); one male, collected at station 717 on the Sedlo Seamount, mounted as a glycerol-paraffin slide on a glass slide (ZMB 11641) (Fig. 1A–D; Table 1).

Additional material for SEM: adults, mounted on aluminum stubs; five males and nine females, collected at station 63 (ZMB 11664a–d, 11665a–d, 11666a, 11667a–d, 11668a), Mediterranean deep sea off Crete; five males and four females, collected at station 66, Mediterranean deep sea off Crete (ZMB 11661a–c, d, 11662a–c, 11663a, c); one male and four females, collected at station 152, the Karasik Seamount (ZMB 11669a, b, 11670b, d, 11671e) (Fig. 1A, B, E; Table 1).

######### Type locality.

Deep-sea trench off Crete, Mediterranean Sea, (34°30'19"N, 26°11'30"E), 4,332 m depth (Fig. 1A, E; Table 1).

######### Description.

**Figure 2.**
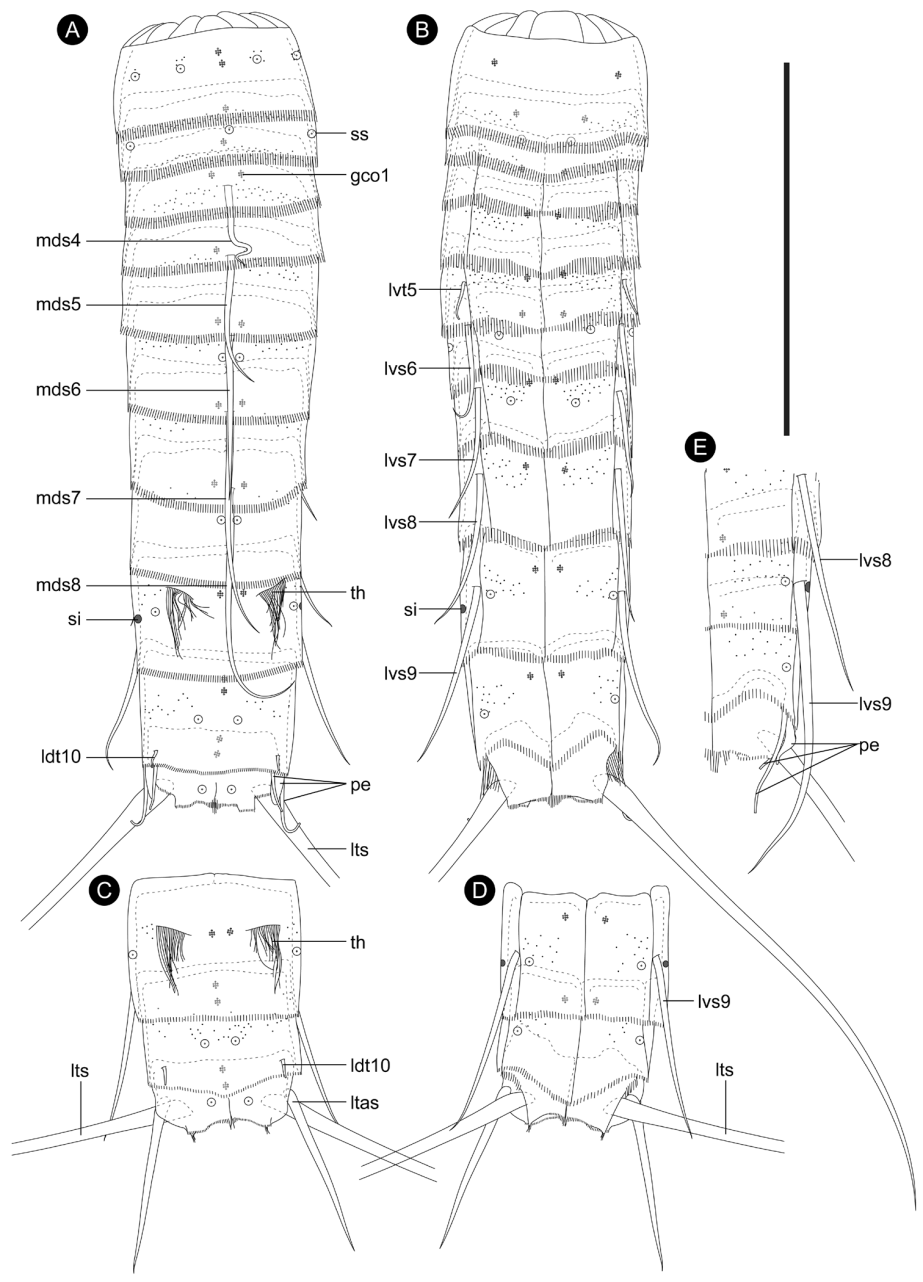
*Echinoderes pterus* sp. n., camera lucida drawings. **A, B** Holotype, male (ZMB 11608), collected at station 55 (Mediterranean deep sea off Crete), entire animal, segments 1–11 in dorsal and ventral view, respectively **C, D** paratype, female (ZMB 11614), collected at station 51 (Mediterranean deep sea off Crete), segments 9–11, dorsal and ventral view, respectively **E** non-type, male (ZMB 11653), collected at station 152-1 (Karasik Seamount), segments 8–11, left side of ventral view. Characters drawn in gray color are overlapped by the preceding segment. Abbreviations: gco1, type-1 gland cell outlet; ldt, laterodorsal tube; ltas, lateral terminal accessory spine; lts, lateral terminal spine; lvs, lateroventral acicular spine; lvt, lateroventral tube; mds, middorsal acicular spine; pe, penile spine; si, protonephridial sieve plate; ss, sensory spot; th, tuft of long hairs. Digits after abbreviations indicate the corresponding segment number except in connection with a gland cell outlet. Scale bar: 100 µm.

**Figure 3.**
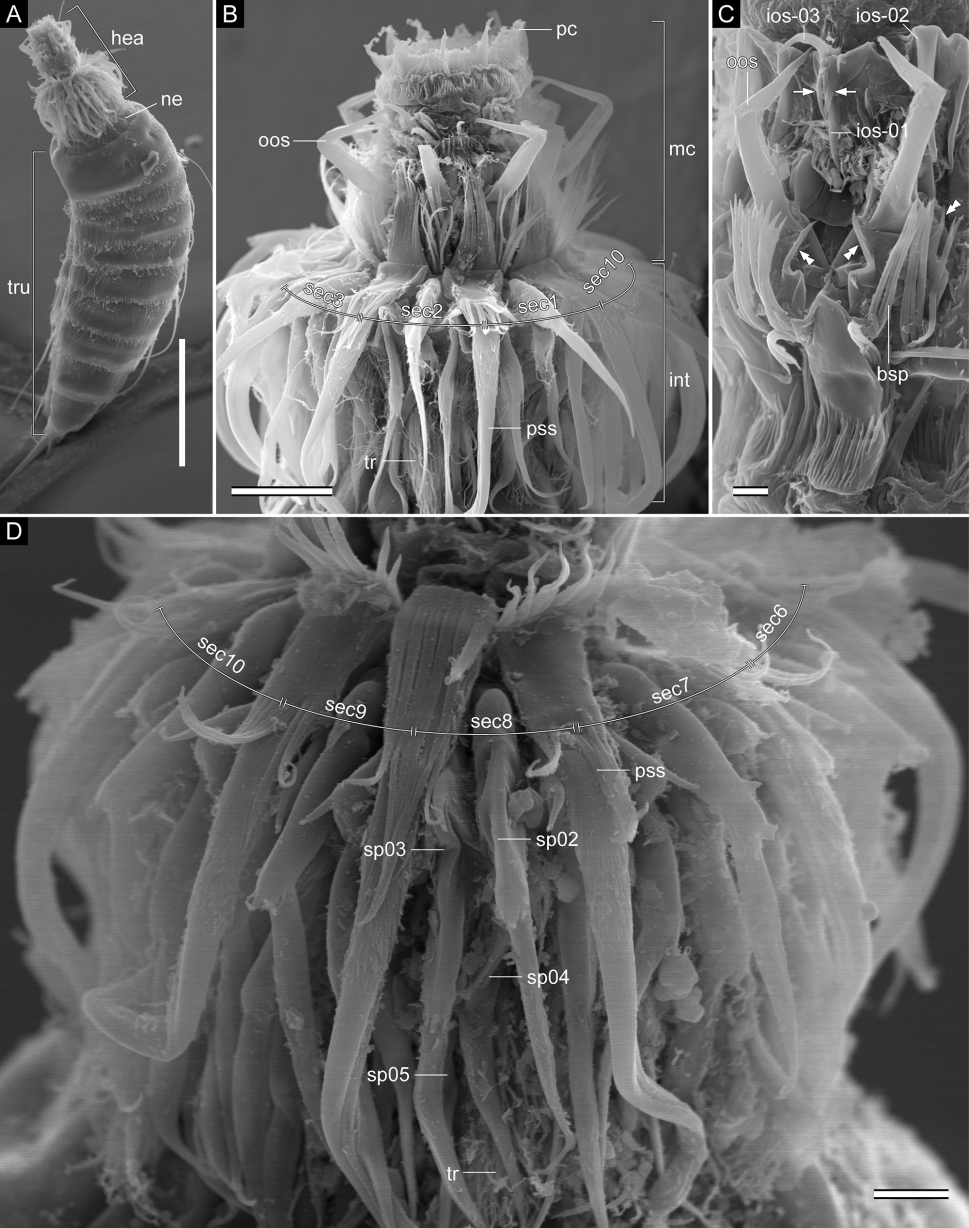
*Echinoderes pterus* sp. n., scanning electron micrographs. Male (**A, D** ZMB 11662c), collected at station 66 (Mediterranean deep sea off Crete) and females (**B** ZMB 11669b; **C** ZMB 11671e), collected at station 152-1 (Karasik Seamount). **A** entire animal, lateral view (left side) **B** head, ventral view **C** close-up of mouth cone, subdorsal view **D** close-up of introvert, lateral view (left side). Abbreviations: bsp, bifurcated spinose processes; hea, head; int, introvert; ios, inner oral style; mc, mouth cone; ne, neck; oos, outer oral style; pc, pharyngeal crown; pss, primary spinoscalid; sec, sector; sp, spinoscalid; tr, trichoscalid; tru, trunk. Digits after abbreviations indicate the sector or ring number. White arrows point to spinose structures at basal part of ring -01 inner oral styles. White double arrowheads indicate short spinose processes. Scale bars: 50 µm (**A**), 10 µm (**B**), 2 µm (**C**), 3 µm (**D**).

**Figure 4.**
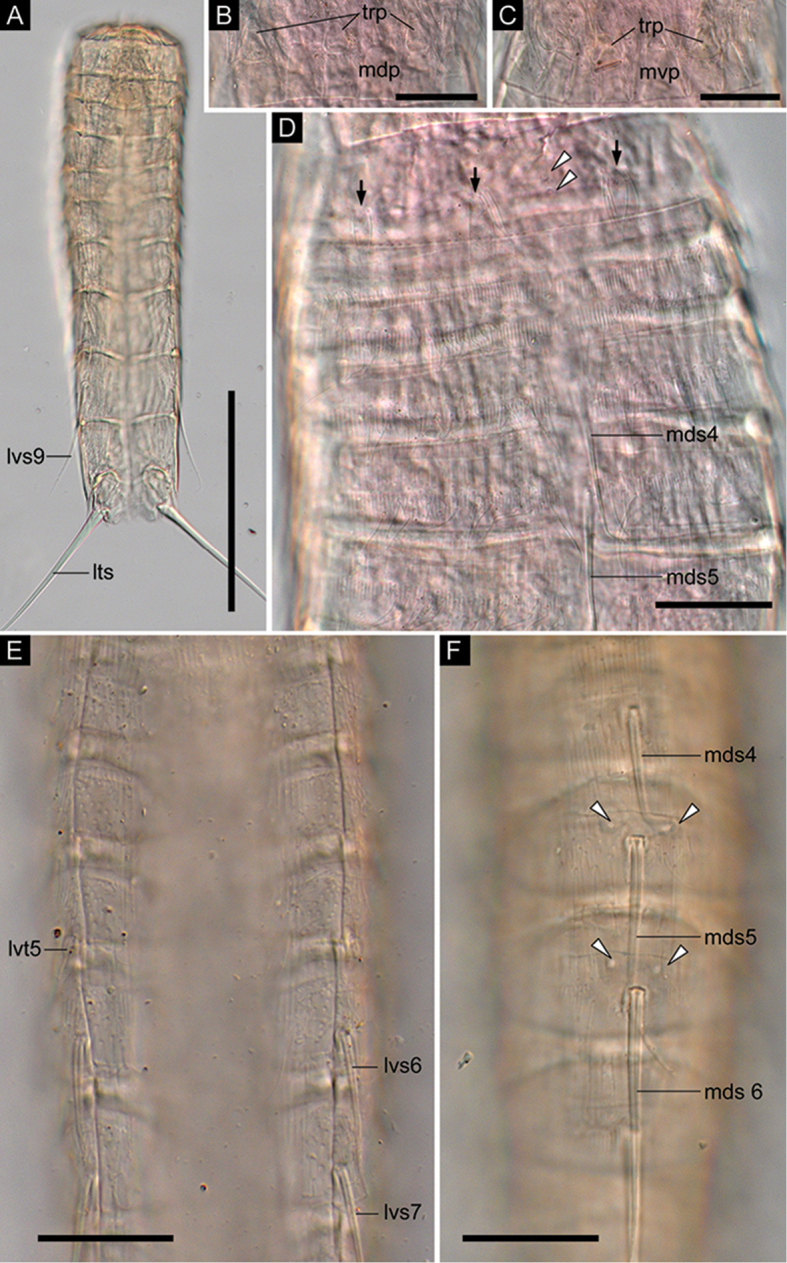
*Echinoderes pterus* sp. n., Nomarski photomicrographs. The holotype male (**A, F** ZMB 11608), collected at station 55 (Mediterranean deep sea off Crete), a male (**E** ZMB 11609), collected at station 24 (Mediterranean deep sea off Crete), and a female (**B–D** ZMB 11635), collected at station 1167.1 (Mediterranean deep sea of Crete). **A** entire animal, ventral view **B** neck, dorsal view **C** neck, ventral view **D** segments 1–6, dorsal view **E** segments 3–7, ventral view **F** segments 4–7, dorsal view. Abbreviations: lts, lateral terminal spine; lvs, lateroventral acicular spine; lvt, lateroventral tube; mdp, middorsal placid; mds, middorsal acicular spine; mvp, midventral placid; trp, trichoscalid plate. Digits after abbreviations indicate the corresponding segment number. Black arrows mark sensory spots; white arrowheads point to type-1 gland cell outlets. Scale bars: 100 µm (**A**), 20 µm (**B–D**).

Adult with head, neck, and eleven trunk segments (Figs 2A, B, 3A, 4A). See Table 2 for measurements. Table 3 indicates the positions of cuticular structures (sensory spots, gland cell outlets, spines, tubes, and sieve plates).

**Table 2.** Measurements of adult *Echinoderes pterus* sp. n. Measurements are given in micrometers, except for the ratios, and are summed for all specimens and listed separately for each population. Columns N and SD indicate sample size and standard deviation, respectively. Abbreviations: (f), length in females; ldt, length of laterodorsal tube; ltas, length of lateral terminal accessory spine; lts, length of lateral terminal spine; lvs, length of lateroventral spine; lvt, length of lateroventral tube; (m), length in males; mds, length of middorsal spine; msw, maximum sternal width; n.a., data not available; s, segment length; sw, standard width; tl, trunk length. Digits after abbreviation indicate segment number.

	Total				Anaximenes Seamount				Mediterranean deep-sea off Crete				Sedlo Seamount		North of Svalbard				Karasik Seamount			
	*N*	Range	Mean	SD	*N*	Range	Mean	SD	*N*	Range	Mean	SD	*N*		*N*	Range	Mean	SD	*N*	Range	Mean	SD
tl	56	186–253	219.3	15.5	3	205–234	215.4	15.9	30	186–253	217.8	16.5	0	n.a.	4	208–241	224.5	14.0	19	196–250	221.2	15.0
msw-5/6	30	42–59	50.2	4.3	2	48–48	47.9	0.3	13	42–54	48.0	3.5	1	49	4	56–59	58.0	1.4	10	46–56	50.5	2.5
msw/tl	29	19–28%	22.9%	2.2%	2	21–23%	21.9%	1.9%	13	19–26%	21.9%	1.7%	0	n.a.	4	23–28%	25.9%	2.1%	10	21–26%	23.2%	1.7%
sw-10	38	35–50	42.8	4.0	2	41–41	41.0	0.3	19	35–49	40.8	4.2	1	41	4	48–50	49.0	0.8	12	41–45	44.2	1.3
sw/tl	37	16–24%	19.7%	1.6%	2	18–20%	18.7%	1.6%	19	16–22%	19.2%	1.6%	0	n.a.	4	21–24%	21.9%	1.2%	12	18–22%	19.9%	1.3%
s1	46	25–38	31.8	2.9	2	29–31	29.9	1.0	25	25–37	31.6	3.1	1	30	4	27–29	28.3	1.0	14	31–38	33.6	1.6
s2	45	16–31	25.2	3.2	2	21–25	22.9	2.9	25	16–31	24.6	3.1	0	n.a.	4	19–23	21.3	1.7	14	23–29	27.6	1.7
s3	44	20–26	23.3	1.4	2	22–23	22.5	0.3	24	20–25	22.6	1.4	0	n.a.	4	23–26	24.5	1.3	14	23–26	24.1	1.1
s4	45	20–30	24.7	1.9	2	24–25	24.1	0.7	25	20–26	23.8	1.4	0	n.a.	4	27–30	28.0	1.4	14	24–27	25.7	1.1
s5	46	22–33	25.8	2.5	2	24–25	24.5	0.7	25	22–27	24.4	1.1	1	24	4	30–33	31.3	1.3	14	25–31	27.3	1.5
s6	46	25–36	28.7	2.5	2	25–28	26.6	2.3	25	25–32	27.4	1.7	1	27	4	32–36	33.8	1.7	14	27–32	29.9	1.4
s7	46	25–38	30.5	2.7	2	29–30	29.2	0.7	25	25–32	28.9	1.5	1	30	4	36–38	36.8	1.0	14	30–33	31.8	1.3
s8	46	29–43	33.8	3.0	2	30–34	31.9	2.6	25	29–37	32.5	2.0	1	30	4	40–43	41.3	1.3	14	32–36	34.4	1.3
s9	46	31–40	34.8	2.2	2	32–34	33.3	1.3	25	31–37	34.0	1.5	1	31	4	39–40	39.8	0.5	14	33–38	35.2	1.5
s10	46	32–50	39.3	4.8	2	32–37	34.5	2.9	25	34–39	36.4	1.4	1	36	4	36–41	38.3	2.2	14	40–50	45.8	3.1
s11	45	19–27	22.8	2.2	2	19–21	19.9	2.0	24	19–27	23.3	1.9	1	19	4	20–25	22.8	2.2	14	19–26	22.6	2.2
mds4	53	25–44	33.1	5.0	3	27–29	28.2	0.9	28	25–36	30.1	3.3	1	35	4	33–36	34.8	1.3	17	33–44	38.5	3.0
mds5	53	34–60	44.5	6.6	3	38–42	39.5	1.9	28	34–47	39.8	3.1	1	50	4	44–50	47.8	2.6	17	45–60	52.1	3.9
mds6	52	44–69	54.0	7.0	3	51–52	51.5	0.8	27	44–57	48.8	3.4	0	n.a.	4	48–60	55.0	5.6	18	56–69	62.0	3.6
mds7	53	56–92	71.4	11.5	3	60–69	63.7	4.6	29	56–69	63.0	3.4	1	68	3	75–76	75.3	0.6	17	79–92	86.6	4.4
mds8	48	71–108	88.0	11.2	2	84–85	84.5	0.3	27	71–88	79.5	4.6	1	101	4	89–99	95.5	4.7	14	95–108	101.9	4.2
ldt10	31	5–13	9.5	2.3	0	n.a.	n.a.	n.a.	12	5–9	7.2	1.3	0	n.a.	0	n.a.	n.a.	n.a.	19	8–13	10.9	1.2
lvt5	49	6–15	9.3	1.8	1	10–10	10.2	n.a.	28	6–15	9.7	2.1	0	n.a.	1	12–12	n.a.	n.a.	19	7–11	8.5	1.0
lvs6	51	22–43	31.7	5.4	3	27–32	29.6	2.3	27	22–38	28.0	3.6	1	35	4	30–35	33.5	2.4	16	32–43	37.7	3.0
lvs7	55	30–49	38.0	4.7	2	32–37	34.5	3.6	29	30–41	34.8	2.9	1	42	4	37–41	39.0	1.8	19	38–49	42.8	3.0
lvs8	56	37–65	48.4	7.0	3	42–46	43.7	2.1	29	37–50	43.9	3.7	1	45	4	47–61	54.5	7.0	19	46–65	55.0	5.2
lvs9	57	49–90	61.9	11.4	3	50–57	54.5	3.6	30	49–64	55.9	3.8	1	83	4	58–88	72.8	15.9	19	55–90	69.1	12.8
lvs9 (m)	25	50–90	66.2	14.9	1	56–56	n.a.	n.a.	15	50–62	55.5	3.1	1	83	2	85–88	86.5	2.1	7	80–90	84.9	3.9
lvs9 (f)	32	49–83	58.6	6.1	2	50–57	53.9	4.9	15	49–64	56.4	4.4	0	n.a.	2	58–60	59.0	1.4	12	55–64	59.9	3.2
lts	56	114–184	154.4	14.5	3	153–155	153.4	1.4	29	114–176	155.8	17.1	1	184	4	138–161	147.0	9.8	19	129–165	152.6	10.4
ltas	25	44–61	54.7	4.4	2	57–61	59.1	2.8	10	44–60	52.4	5.1	0	n.a.	2	51–52	51.5	0.7	11	52–61	56.7	2.5
lts/tl	55	52–86%	70.5%	7.2%	3	65–75%	71.5%	5.3%	29	57–86%	71.9%	8.2%	0	n.a.	4	63–69%	65.5%	3.1%	19	52–78%	69.3%	5.9%
ltas/tl	25	20–29%	25.2%	2.5%	2	28–29%	28.7%	1.1%	10	20–28%	23.9%	2.4%	0	n.a.	2	22–25%	23.6%	1.9%	11	22–29%	26.0%	2.1%

**Table 3.** Summary of locations of cuticular structures and appendages in *Echinoderes pterus* sp. n. Underlined structure was observed only in specimens from the Anaximenes Seamount and the Mediterranean deep sea off Crete. Abbreviations: ac, acicular spine; (f), female condition of sexually dimorphic character; gco1, type-1 gland cell outlet; la, lateral accessory; ld, laterodorsal; ltas, lateral terminal accessory spine; lts, lateral terminal spine; lv, lateroventral; (m), male condition of sexually dimorphic character; md, middorsal; ml, midlateral; pd, paradorsal; pe, penile spine; sd, subdorsal; si, sieve plate; sl, sublateral; ss, sensory spot; tu, tube; vl, ventrolateral; vm, ventromedial.

Position	md	pd	sd	ld	ml	sl	la	lv	vl	vm
segment										
1	gco1, gco1		ss	ss				gco1		
2	gco1, ss			ss						gco1, ss
3	gco1									gco1
4	ac	gco1								gco1
5	ac	gco1						tu		gco1
6	ac	gco1, ss			ss			ac		ss, gco1
7	ac	gco1						ac		ss, gco1
8	ac	gco1, ss						ac		gco1
9		gco1		ss		si		ac	ss	gco1
10	gco1, gco1		ss	tu					ss	gco1
11	gco1, gco1		ss				pe×3 (m), ltas (f)	lts		

**Figure 5.**
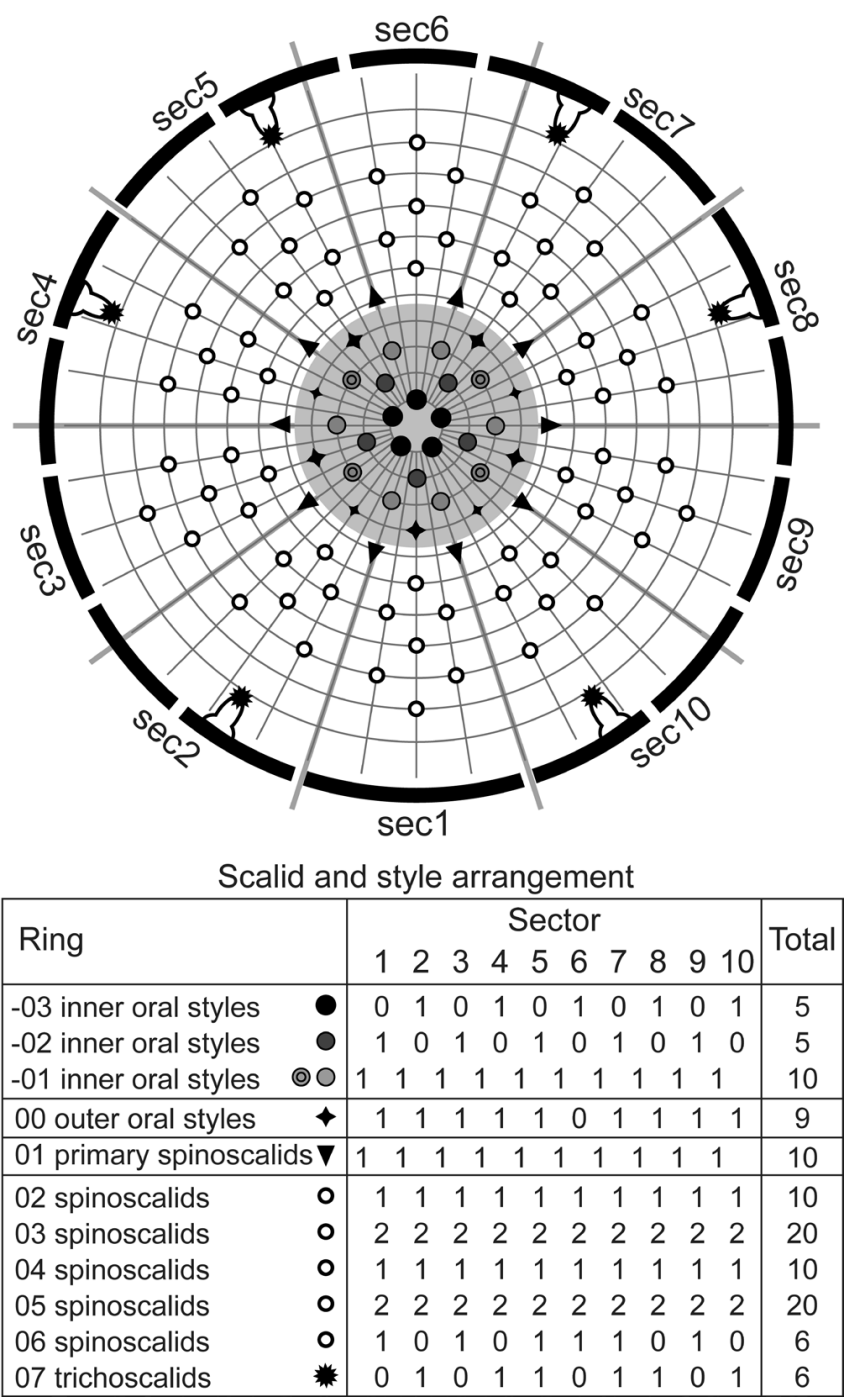
Polar-coordinate diagram of mouth cone, introvert, and placids in *Echinoderes pterus* sp. n. Grey area and heavy line arcs show mouth cone and placids respectively. The table lists the arrangement of styles and scalids by sector. Inner oral styles of ring 01 showing spinose processes at basal part indicated by black circle in grey outer circle. Abbreviation: sec, sector.

Head consisting of retractable mouth cone and introvert (Fig. 3B). Mouth cone with three rings of inner oral styles and nine outer oral styles (Figs 3C, 5). Pharyngeal crown shown anterior to inner oral styles in specimens with artificially protruded head (Fig. 3B), but located interior and posterior of inner oral styles in nature. Five thin and tube-like inner oral styles in ring 03, five thick spinose inner oral styles in ring 02, and ten spinose inner oral styles in ring 01. Two spinose structures present at basal part of ring -01 inner oral styles between sectors 2 and 3, 4 and 5, 7 and 8, and 9 and 10 (Figs 3C, 5). Each outer oral style consisting of rectangular basal part and triangular distal part, with basal part alternating in size between five larger ones in odd sectors and four smaller ones in even sectors (Figs 3B, C, 5). Each outer oral style with six long spinose processes bifurcated at their tips. One pair of additional short spinose processes originating slightly more anteriorly and laterally on either side of each outer oral style. Introvert composed of one ring of primary scalids, five rings of spinoscalids, and one ring of trichoscalids (Figs 3B, D, 5). Each primary spinoscalid consisting of basal sheath and distal end piece. Basal sheath with two layers of proximal fringes. End piece long, covered with minute hairs proximally, bluntly ending at distal tip. Each spinoscalid of rings 02–05 composed of basal sheath with fringed edge and distal long-spinose end piece. Spinoscalids in rings 02 and 03 longer than those in rings 04 and 05. Thin hair-like structures present at basal part of each spinoscalid. Trichoscalids arising from trichoscalid plates. Each trichoscalid covered with long hairs.

Neck with 16 placids (Figs 2A, B, 4B, C). Midventral placid broadest (Fig. 4C). Remaining placids similar in size. Two trichoscalid plates present ventrally and four dorsally, each associated with ventromedial, subdorsal, and laterodorsal placid, respectively (Fig. 4B, C).

**Figure 6.**
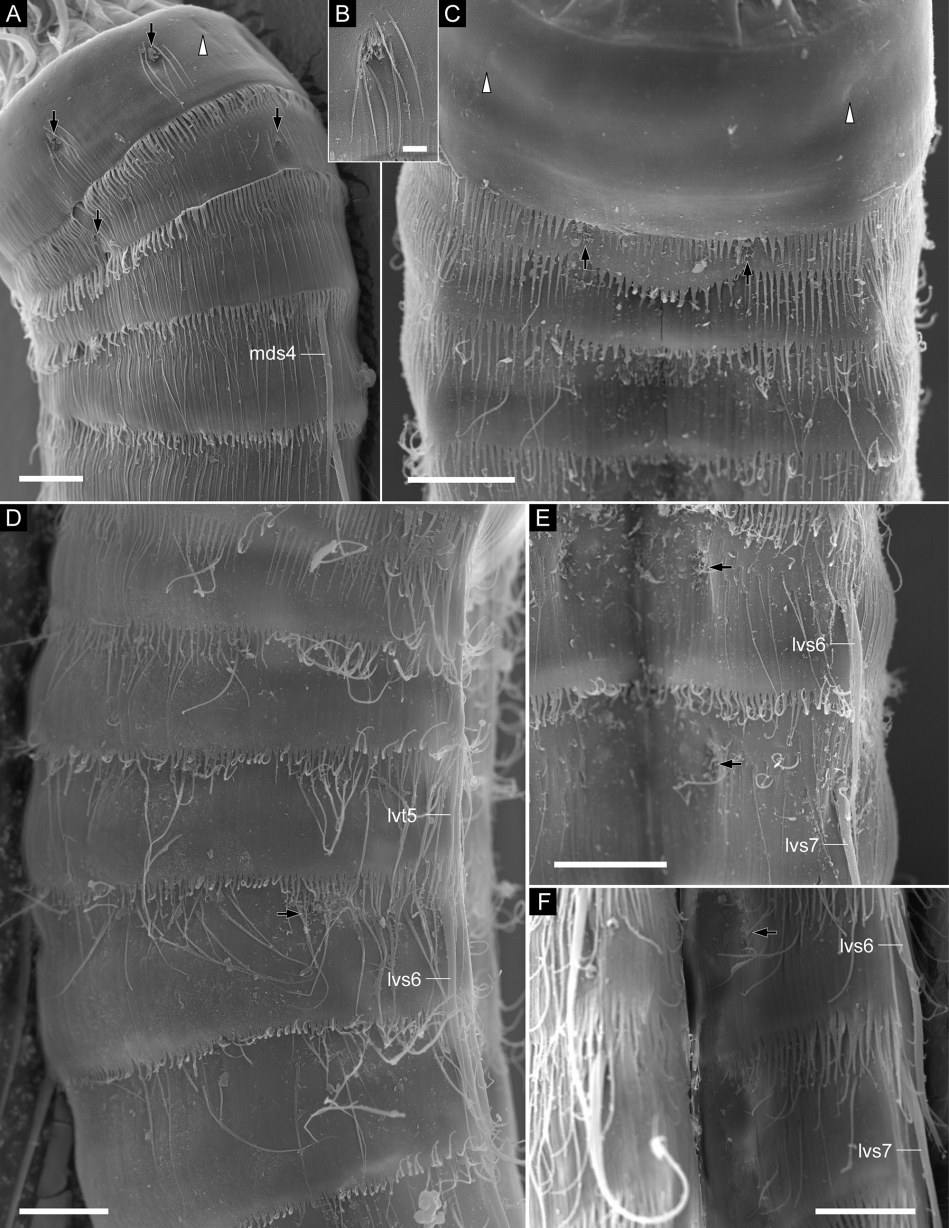
*Echinoderes pterus* sp. n., scanning electron micrographs. Females (**A, B** ZMB 11669a, collected at station 152-1 (Karasik Seamount) **D** ZMB 11661c, collected at station 66 (Mediterranean deep sea of Crete) **F** ZMB 11669b, collected at station 152-1 (Karasik Seamount)) and a male (**C, E** ZMB 11661a, collected at station 66 (Mediterranean deep sea off Crete)). **A** segments 1–4, laterodorsal view (left side) **B** close-up of laterodorsal sensory spot on segment 1 **C** segments 1–4, ventral view **D** segments 3–7, lateral view (right side) **E** sternal plates on segments 6 and 7 **F** sternal plates on segments 6 and 7. Abbreviations: lvs, lateroventral acicular spine; lvt, lateroventral tube; mds, middorsal acicular spine. Digits after abbreviations indicate the corresponding segment number. Black arrows point to sensory spots; white arrowheads mark type-1 gland cell outlets. Scale bars: 10 µm (**A, C–F**), 2 µm (**B**).

Segment 1 consisting of complete cuticular ring. Sensory spots located in subdorsal and laterodorsal position (Figs 2A, 4D, 6A, B). Few hairs flanking each sensory spot. Two type-1 gland cell outlets present in tandem in middorsal and additional single pair in lateroventral position (Figs 2A, B, 4D, 6A, C). Posterior part of this and following ten segments with primary pectinate fringe (Figs 2A, B, 4D, 6A, C). Pectinate fringe teeth of primary pectinate fringe thin and long. Segment devoid of cuticular hairs except for hairs associated with sensory spots (Figs 3A, 6A, C).

**Figure 7.**
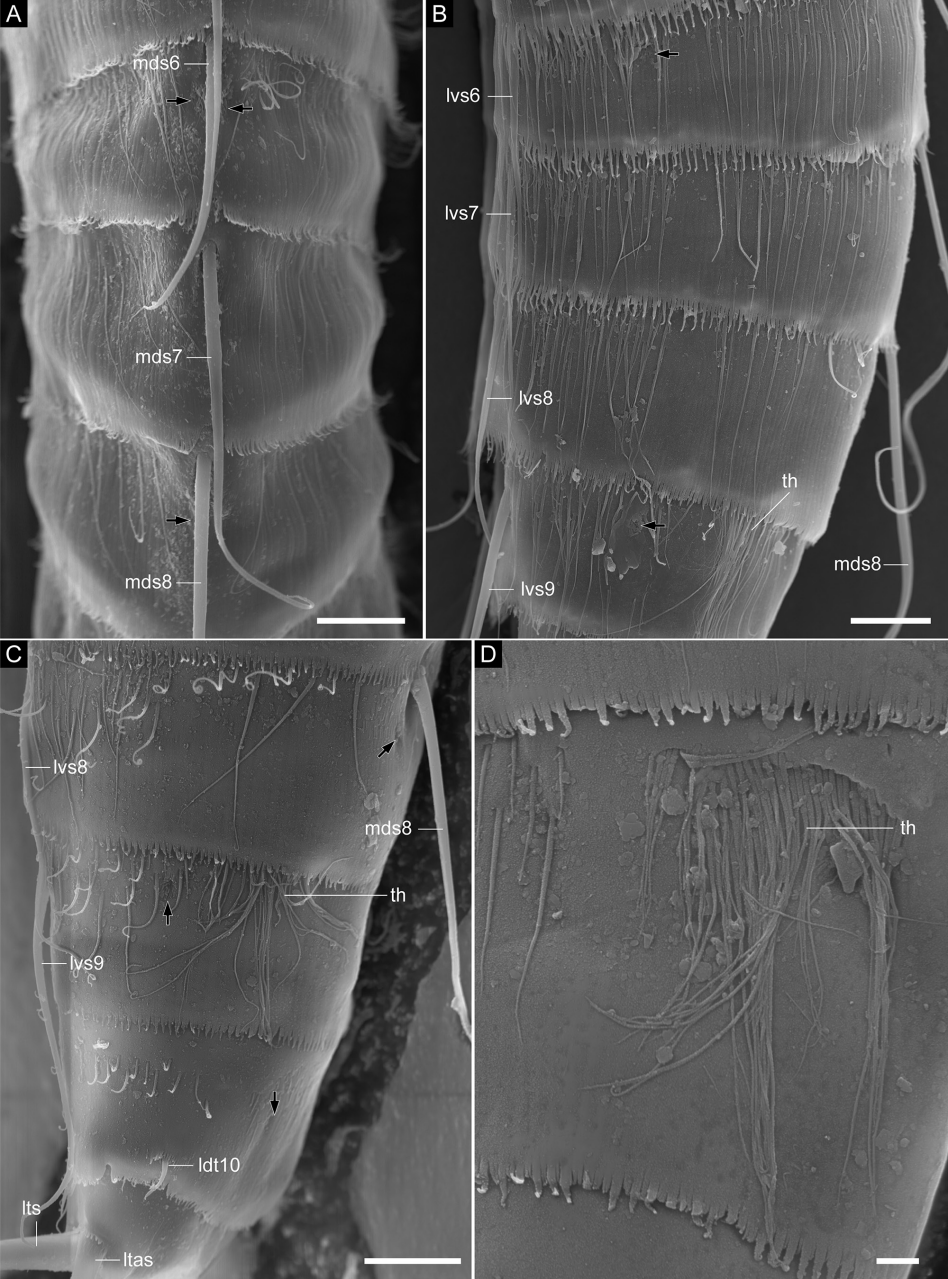
*Echinoderes pterus* sp. n., scanning electron micrographs. Females (**A** ZMB 11661b, collected at station 66 (Mediterranean deep sea off Crete) **B** ZMB 11669a, collected at station 152-1 (Karasik Seamount) **C** ZMB 11664a, collected at station 63 (Mediterranean deep sea off Crete) **D** ZMB 11665d, collected at station 63 (Mediterranean deep sea off Crete)). **A** segments 6–8, dorsal view **B** segments 6–9, lateral view (left side) **C** segments 8–11, lateral view (left side) **D** close-up of tuft of hairs on segment 9. Abbreviations: ldt, laterodorsal tube; ltas, lateral terminal accessory spine; lts, lateral terminal spine; lvs, lateroventral acicular spine; mds, middorsal acicular spine; th, tuft of long hairs. Digits after abbreviations indicate the corresponding segment number. Black arrows point to sensory spots. Scale bars: 10 µm (**A–C**), 2 µm (**D**).

Segment 2 with complete cuticular ring as segment 1. This and following eight segments with thick pachycyclus at anterior margin of each segment (Figs 2A, B, 4A, D–F). Pachycyclus interrupted middorsally in segments 3–9 as well as at tergosternal and midsternal junctions in segments 3–10. Cuticular hairs rising from perforation sites in anterior and central area of this and following eight segments (Fig. 6A); hairs long, rather thin and flexible, and tending to curl up (Figs 6D, 7C). Sensory spots present in middorsal, laterodorsal and ventromedial position (Figs 2A, B, 6A, C). Type-1 gland cell outlets present in middorsal and ventromedial position.

Segment 3 and following eight segments consisting of one tergal and two sternal plates (Fig. 2A, B). No sensory spots present. Type-1 gland cell outlets situated in middorsal and ventromedial position.

Segment 4 with middorsal acicular spine (Figs 2A, 4D, D, 6A). No sensory spots present. Type-1 gland cell outlets present in paradorsal and ventromedial position.

Segment 5 with middorsal acicular spine and lateroventral tubes (Figs 2A, B, 4D–F, 6D). Lateroventral tubes consisting of relatively thick and short basal part and long flexible distal part. Sensory spots absent. Type-1 gland cell outlets present in paradorsal and ventromedial position (Fig. 4F).

Segment 6 with middorsal and lateroventral acicular spines (Figs 2A, B, 4E, F, 6D–F, 7A, B). Sensory spots present in paradorsal, midlateral, and ventromedial position (Figs 2A, B, 6D–F, 7A, B). Type-1 gland cell outlets present paradorsally and ventromedially (Fig. 4F).

**Figure 8.**
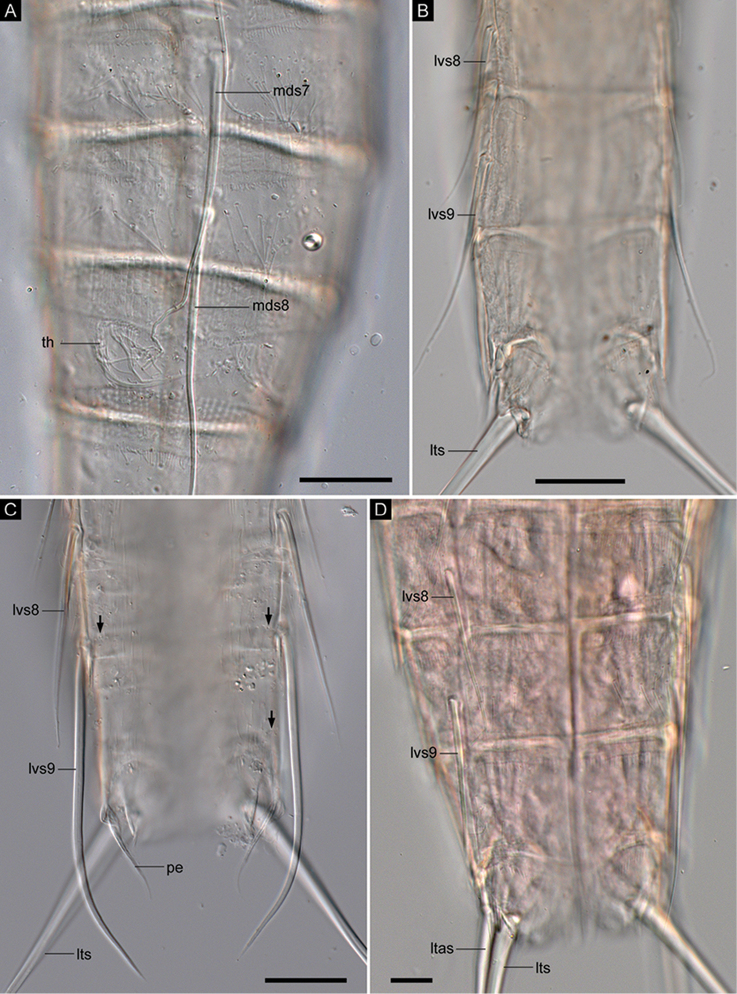
*Echinoderes pterus* sp. n., Nomarski photomicrographs. The holotype male (**B** ZMB 11608, collected at station 55, Mediterranean deep sea off Crete), non-type males (**A** ZMB 11641, collected at station 717, Sedlo Seamount **C** ZMB 11653, collected at station 152-1, Karasik Seamount), and a female (**D** ZMB 11635, collected at station 1167.1, Mediterranean deep sea off Crete). **A** segments 7–9, dorsal view **B** segments 8–11, ventral view **C** segments 8–11, ventral view **D** segments 8–11, ventral view. Abbreviations: ltas, lateral terminal accessory spine; lts, lateral terminal spine; lvs, lateroventral acicular spine; mds, middorsal acicular spine; pe, penile spine; th, tuft of long hairs. Digits after abbreviations indicate the corresponding segment number. Black arrows point to sensory spots. Scale bars: 20 µm (**A–C**), 10 µm (**D**).

Segment 7 with middorsal and lateroventral acicular spines (Figs 2A, B, 4E, 6E, 7A, B, 8A). Sensory spots present in ventromedial position in specimens from Mediterranean deep sea off Crete and those from the Anaximenes Seamount (Fig. 6E). Sensory spots absent in specimens from the Karasik Seamount, north of Svalbard, and the Sedlo Seamount (Fig. 6F). Type-1 gland cell outlets present paradorsally and ventromedially.

**Figure 9.**
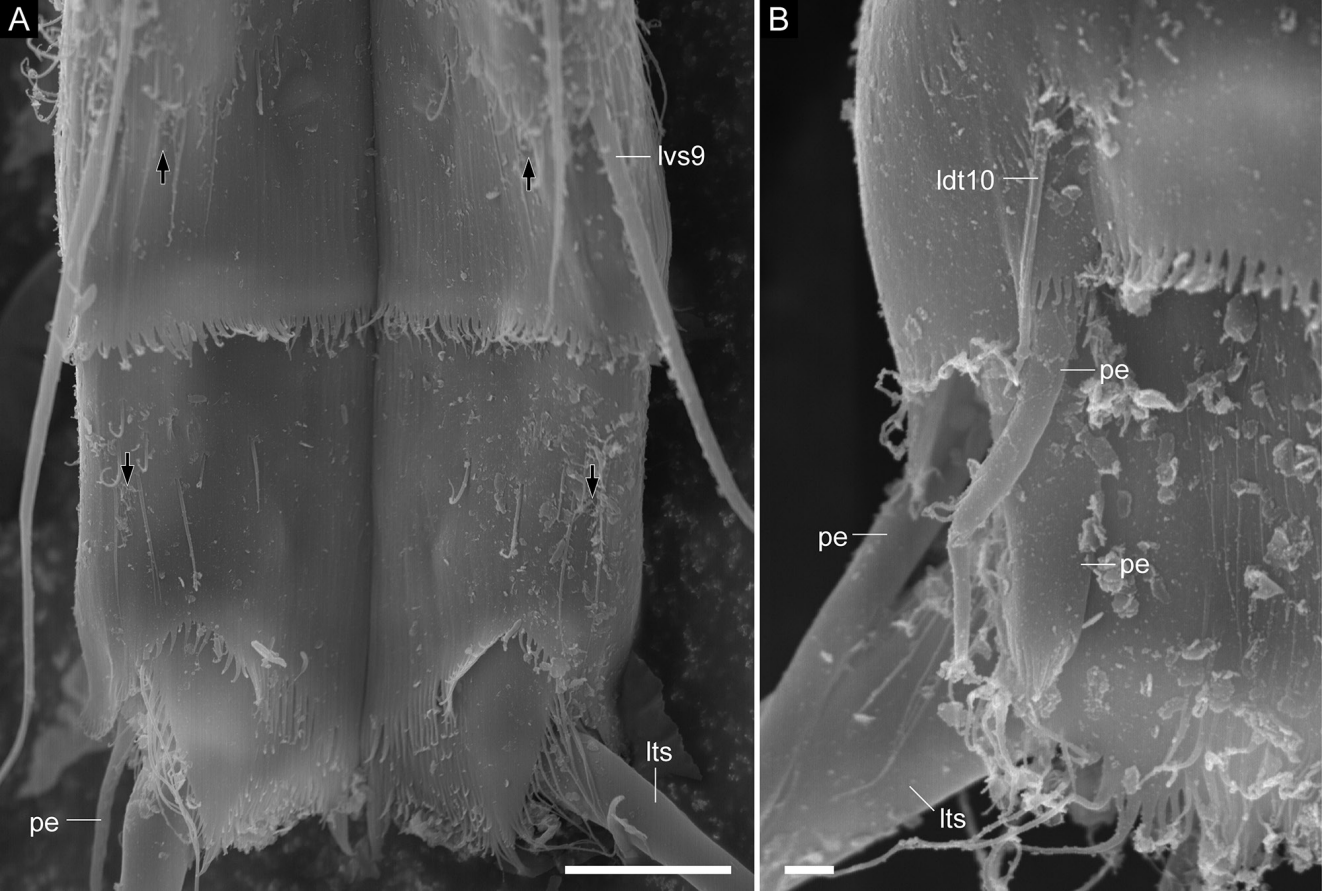
*Echinoderes pterus* sp. n., scanning electron micrographs. Males (**A** ZMB 11662b **B** ZMB 11662a, both collected at station 66 (Mediterranean deep sea off Crete)). **A** segments 9–11, ventral view **B** left side of sternal plates on segments 10 and 11. Abbreviations: ldt, laterodorsal tube; lts, lateral terminal spine; lvs, lateroventral acicular spine; pe, penile spine. Digits after abbreviations indicate the corresponding segment number. Black arrows point to sensory spots. Scale bars: 10 µm (**A**), 1 µm (**B**).

Segment 8 with middorsal and lateroventral acicular spines (Figs 2A, B, E, 7A–C, 9A–D). Sensory spots present paradorsally (Fig. 7A, C). Type-1 gland cell outlets present in paradorsal and ventromedial position.

Segment 9 with lateroventral acicular spines (Figs 2B, D, E, 4A, 7B, C, 8B–D, 9A). Lateroventral acicular spines in male specimens from the Karasik Seamount, north of Svalbard, and the Sedlo Seamount conspicuously thick and long (Figs 2E, 8C), whereas thickness of spines similar to those on preceding segments in other specimens (Figs 2B, D, 4A, 7B, C, 8B, D, 9A). Tufts of hairs arising from slits in laterodorsal position (Figs 2A, C, 7B–D, 8A). Most hairs of the tufts conspicuously longer than other usual cuticular hairs. Laterodorsal and ventrolateral sensory spots present (Figs 7B, C, 8C, 9A). Type-1 gland cell outlets present in paradorsal and ventromedial position. Small rounded sieve plates present in sublateral position.

Segment 10 with laterodorsal tubes (Figs 2A, C, 7C, 9B). Subdorsal and ventrolateral sensory spots present (Figs 7C, 8C, 9A). Two type-1 gland cell outlets aligned middorsally. Additional pair of type-1 gland cell outlets present in ventromedial position.

Segment 11 with lateral terminal spines (Figs 2A–D, 4A, 7C, 8B–D). Three pairs of penile spines present in males, with two pairs being tube-like and one pair thick and cone shaped (Figs 2A, E, 8C, 9A, B). One pair of lateral terminal accessory spines present in females (Figs 2C, D, 7C, 8D). Subdorsal sensory spots present. Two type-1 gland cell outlets present middorsally. Tergal extensions very short and truncate; sternal extensions triangular, extending slightly beyond tergal ones (Figs 2A–D, 4A, 8B, D, 9A, B).

### Differential diagnosis

*Echinoderes pterus* sp. n. can be easily distinguished from all the other congeners by the presence of the tufts of hairs on segment 9. Such a structure has never been described for any other kinorhynch, and is thus a unique character for the new species. This is also the case for the conspicuously thick and long lateroventral spines on segment 9, although this character appears to be restricted to males in the Karasik Seamount, Svalbard, and the Sedlo Seamount populations.

With respect to other characters, the spine/tube pattern of *E. pterus* sp. n., i.e., with middorsal acicular spines on segments 4–8, laterodorsal tubes on segment 10, lateroventral tubes on segment 5, and lateroventral acicular spines on segments 6–9, but without any other spine and tube is not shared with any of 109 congeners.

The head morphology of *E. pterus* sp. n. seems to be shared with only a few species of Kinorhyncha. In the new species, the ring -02 and -03 inner oral styles occur in odd and even sectors, respectively. Such an arrangement is known for *Dracoderes abei* Higgins & Shirayama, 1990 (see Sørensen et al. 2012a), whereas the position of these styles seems to be reversed in all cyclorhagid species for which the arrangement of the inner oral styles is known, i.e., *Antygomonas caeciliae* Dal Zotto, 2015, *Antygomonas incomitata* Nebelsick, 1990, *Antygomonas oreas* Bauer-Nebelsick, 1996, *Antygomonas paulae* Sørensen, 2007, *Cateria gerlachi* Higgins, 1968, *Ce. barbanigra*, *Centroderes bonnyae* Neuhaus et al., 2014, *Centroderes drakei* Neuhaus et al., 2014, *Centroderes readae* Neuhaus et al., 2014, *Cephalorhyncha liticola* Sørensen, 2008, *Semnoderes armiger* Zelinka, 1928 *Tubulideres seminoli* Sørensen et al., 2007, *Triodontoderes anulap* Sørensen & Rho, 2009 (see Bauer-Nebelsick 1996; Sørensen 2007, 2008; Sørensen et al. 2007, 2009; Sørensen and Rho 2009; Dal Zotto 2015; Neuhaus et al. 2014; Neuhaus and Kegel 2015). However in *Cat. gerlachi*, only a single specimen mounted for light microscopy had its inner oral styles everted enough to be recognizable, and the mouth cone was separated from the specimen (Neuhaus and Yamasaki, unpubl. obs.). Neuhaus and Kegel (2015, fig. 3) illustrated ring -02 and -03 inner oral styles in the position they assumed to be correct. This raises the question how accurate identification of the position was in other species by these and other authors. Since the exact arrangements and the shapes of inner oral styles have been infrequently observed in *Echinoderes*, it is not possible to conclude whether those in *E. pterus* sp. n. are unique among the genus or not. We hope that further observations of head structures in other species of *Echinoderes* will allow a comprehensive comparison of this character in the future.

## Discussion

### Geographically and bathymetrically wide distribution in Kinorhyncha

*Echinoderes pterus* sp. n. shows a geographically and bathymetrically wide distribution, from near the North Pole to the eastern Mediterranean Sea through the northeast Atlantic Ocean, and from 675 m to 4,403 m depth (Fig. 1, Table 1). For other kinorhynchs, such a geographically and bathymetrically wide distribution is only known for *Cam. vanhoeffeni*, *Centroderes spinosus* (Reinhard, 1881), and *S. armiger*. The former one was reported worldwide at a depth ranging from 0–5,118 m from several localities in the Atlantic Ocean, Pacific Ocean, Indian Ocean, and the Antarctic Sea (Neuhaus and Sørensen 2013). The latter two were found in the Mediterranean Sea, Black Sea, north-eastern Atlantic Ocean, and North Sea at depths ranging from 14 m to 444 m (*Ce. spinosus*) and from 15 m to 444 m (*S. armiger*) (Neuhaus 2013; Neuhaus et al. 2013).

There are few other kinorhynchs which have been reported to show either a geographically or a bathymetrically wide distribution. Species with a geographically wide distribution are e.g., *Ce. barbanigra* found in the Gulf of Mexico, the Caribbean Sea, Bermuda, and the Dominican Republic at a depth ranging from 2 m to 57.5 m, *E. ohtsukai* found on both the eastern and western coasts of the Pacific Ocean in the intertidal zone, and *E. tchefouensis* found in the East China Sea, South China Sea, Celebes Sea, Singapore Strait, and Mariana Islands at a depth ranging from 0 m to 140 m (Sørensen et al. 2012b, 2016; Yamasaki and Kajihara 2012; Neuhaus et al. 2014; Herranz and Leander 2016). Species from a bathymetrically wide range are e.g., *Echinoderes arlis* Higgins, 1966, *Echinoderes drogoni* Grzelak & Sørensen, 2018, *Echinoderes eximus* Higgins & Kristensen, 1988, *Echinoderes peterseni* Higgins & Kristensen, 1988, and *Echinoderes rhaegali* Grzelak & Sørensen, 2018, all found in the Arctic Ocean, at depths ranging from 236 m to 940 m (*E. arlis*), 78 m to 2,200 m (*E. drogoni*), 60 m to 940 m (*E. eximus*), 24 m to 940 m (*E. peterseni*), and 78 m to 940 m (*E. rhaegali*) (Grzelak and Sørensen 2018b). However, their distribution records are not both geographically and bathymetrically wide like those of *E. pterus* sp. n., *Cam. vanhoeffeni*, *Ce. spinosus*, and *S. armiger*.

### A single or multiple species?

**Figure 10.**
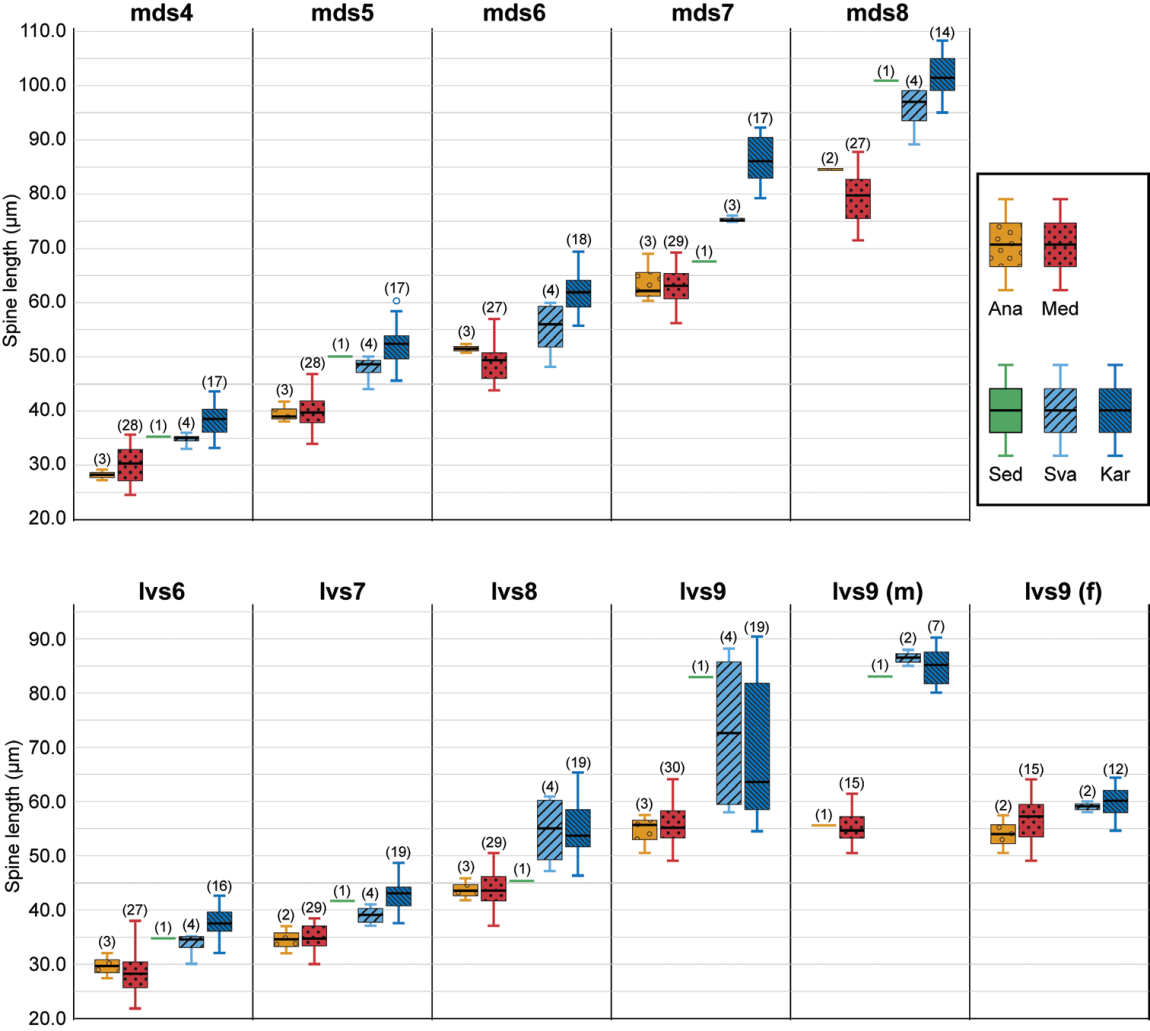
Box plot for the spine-lengths comparison among populations of *Echinoderes pterus* sp. n. Each color represents one population. The numbers above a box indicate the number of measured specimens for each character and population. Abbreviations: Ana, population from the Anaximenes Seamount; (f), length in females; Kar, population from the Karasik Seamount; lvs, lateroventral acicular spine; (m), length in males; Med, population in the Mediterranean deep sea off Crete; mds, middorsal acicular spine; Sed, population from the Sedlo Seamount; Sva, population north of Svalbard. Digits after mds and lvs indicate the corresponding segment number.

The morphological comparison between populations of *E. pterus* sp. n. reveals that the new species shows an inter-population variation (Fig. 10, Table 2). The most obvious difference is found between males of the Arctic populations (Karasik Seamount + Svalbard) + the Sedlo Seamount population with lateroventral acicular spines on segment 9 being conspicuously thicker and longer than the preceding spines, as opposed to those of the Mediterranean populations (Mediterranean deep sea + Anaximenes Seamount), which have lateroventral spines on segment 9 of similar thickness to the other spines and only slightly longer than the preceding ones (compare Fig. 8B and Fig. 8C; see Fig. 10 for measurements). Such a large difference is not found between females of these populations. In addition, the length of the remaining spines is slightly longer in the Arctic populations than in the Mediterranean populations (Fig. 10). The population on the Sedlo Seamount, although it is represented by a single specimen in this study, shows similarities in spine length to the Arctic populations in the middorsal acicular spines on segments 4, 5, 8 and the lateroventral acicular spines on segments 6, 7, 9, whereas it shares a similar spine length with the Mediterranean populations in the other spines. The ventromedial sensory spots on segment 7 reveal variation insofar as they are absent in the Arctic and Sedlo Seamount populations but present in the Mediterranean populations.

Considering the geographically and bathymetrically wide distribution of *E. pterus* sp. n., the presence of inter-population variation in morphological characters, as well as the potentially low-distribution ability of kinorhynchs, it should be considered whether *E. pterus* sp. n. represents one or multiple species. In the case of the other geographically and bathymetrically wide distribution kinorhynchs, intra- and inter-populational variation of several morphological characters, e.g., body length, arrangement of gland cell outlets, and sensory spots, has been detected in *Cam. vanhoeffeni*. However, it was still regarded as a single species due to the overlapping characters between/within populations and the absence of the type material (Neuhaus and Sørensen 2013). Variation in the occurrence of sensory spots within one species has also been reported for several other kinorhynchs, e.g., *Cat. gerlachi*, *Cateria styx* Higgins, 1968, *Ce. spinosus*, *Ce. barbanigra*, and *Ce. readae* (Neuhaus et al. 2013, 2014; Neuhaus and Sørensen 2013; Neuhaus and Kegel 2015).

*Echinoderes pterus* sp. n. may on the one hand represent two species, e.g., one species in the Arctic Ocean and on the Sedlo Seamount and the second species in the Mediterranean, or it may even belong to three species, i.e., one in the Arctic Ocean, another on the Sedlo Seamount, and the third in the Mediterranean, with only a few morphological differences. However, there is the possibility that the different populations belong to the same species with the observed morphological variations, which gradually change from the Arctic Ocean via the Sedlo Seamount to the Mediterranean populations or *vice versa*. Although we cannot reject these possibilities, we currently regard all populations as a single species. Further investigations of the species, for instance the sampling and observation of populations in intermediate localities and/or molecular phylogeographic studies, should provide more information about the population connectivity of the species and support one of the two hypotheses.

Whichever hypothesis is correct, all populations in this study are undoubtedly closely related to each other. They have expanded their habitat range with or without speciation, however, their distribution process is open to question: did they distribute from the Arctic Ocean via the Atlantic Ocean to the Mediterranean, from the Mediterranean via the Atlantic Ocean to the Arctic Ocean, or from the Atlantic Ocean to both the Arctic Ocean and the Mediterranean? Indeed the species represents interesting material for studying the “meiofauna paradox” or the “everything is everywhere hypothesis”. We cannot provide a strongly-supported answer based on our current data. Further data about the species distribution range and population connectivity would also enable us to approach the question in future studies.

## Supplementary Material

XML Treatment for Echinoderes pterus
